# Single-Cell Photothermal Analysis Induced by MoS_2_ Nanoparticles by Raman Spectroscopy

**DOI:** 10.3389/fbioe.2022.844011

**Published:** 2022-03-10

**Authors:** Giulia Rusciano, Angela Capaccio, Antonio Sasso, Manjot Singh, Mohammadhassan Valadan, Carmela Dell’Aversana, Lucia Altucci, Carlo Altucci

**Affiliations:** ^1^ Department of Physics “E. Pancini”, University of Naples Federico II, Naples, Italy; ^2^ CNR-INO, National Research Council—National Institute of Optics, Pozzuoli, Italy; ^3^ Department of Advanced Biomedical Sciences, University of Naples Federico II, Naples, Italy; ^4^ CNR-IEOS, National Research Council—Institute of Experimental Endocrinology and Oncology—IEOS, Naples, Italy; ^5^ Department of Precision Medicine, University of Campania “Luigi Vanvitelli”, Naples, Italy; ^6^ BIOGEM, Biologia e Genetica Molecolare, Ariano Irpino, Italy; ^7^ INFN Sezione di Napoli, Compl. Univ. di Monte S. Angelo, Napoli, Italy

**Keywords:** MoS_2_, nanosheet, photothermal therapy, single-cell Raman analysis, temperature profiling in single cells

## Abstract

Two-dimensional nanomaterials, such as MoS_2_ nanosheets, have been attracting increasing attention in cancer diagnosis and treatment, thanks to their peculiar physical and chemical properties. Although the mechanisms which regulate the interaction between these nanomaterials and cells are not yet completely understood, many studies have proved their efficient use in the photothermal treatment of cancer, and the response to MoS_2_ nanosheets at the single-cell level is less investigated. Clearly, this information can help in shedding light on the subtle cellular mechanisms ruling the interaction of this 2D material with cells and, eventually, to its cytotoxicity. In this study, we use confocal micro-Raman spectroscopy to reconstruct the thermal map of single cells targeted with MoS_2_ under continuous laser irradiation. The experiment is performed by analyzing the water O-H stretching band around 3,400 cm^−1^ whose tetrahedral structure is sensitive to the molecular environment and temperature. Compared to fluorescence-based approaches, this Raman-based strategy for temperature measurement does not suffer fluorophore instability, which can be significant under continuous laser irradiation. We demonstrate that irradiation of human breast cancer MCF7 cells targeted with MoS_2_ nanosheets causes a relevant photothermal effect, which is particularly high in the presence of MoS_2_ nanosheet aggregates. Laser-induced heating is strongly localized near such particles which, in turn, tend to accumulate near the cytoplasmic membrane. Globally, our experimental outcomes are expected to be important for tuning the nanosheet fabrication process.

## 1 Introduction

Two-dimensional nanomaterials (2D-NM) are an intriguing class of materials exhibiting a sheet-like structure and peculiar physical and chemical properties, which arise when the thickness of bulk materials approaches the single layer (Tan et al. (2017); Fang et al. (2020)). Boosted by the success of graphene, 2D-NM have recently found applications in many fields (Molaei (2021)), including electronics, sensors, catalysis, and biomedicine (Nguyen et al. (2020)). In this frame, transition metal dichalcogenides (TMDs) play a fundamental role (Manzeli et al. (2017)). They exhibit a direct bandgap in the monolayer form, which makes them particularly attractive for optoelectronics applications (Yin et al. (2021)). In particular, MoS_2_ nanosheets (2D-MoS_2_) exhibit a strong photoluminescence emission in the red region, rendering them suitable as labels in biological investigations. Functionalized 2D-MoS_2_ have also been studied with different human cell lines to shed light on their potential in drug delivery applications and cancer diagnosis (Zhang et al. (2017); Liu et al. (2018); Teo et al. (2014)). Moreover, thanks to the strong absorbance in the near-infrared region where most of the tissues are transparent (*NIR water window*), many research groups have tested 2D-MoS_2_ as photothermal agents (PTAs) for cancer therapy, also in combination with photochemical therapy and chemotherapy (Chou et al. (2013); Carrow et al. (2020)). Nowadays, the use of 2D-MoS_2_ for photothermal therapy (PTT) has been well assessed (Wang et al. (2021)). Numerous groups have reported on the effectiveness of properly functionalized 2D-MoS_2_ for killing targeted tissues upon irradiation with NIR radiation, in both *in vitro* and *in vivo* experiments (Liu et al. (2021); Song et al. (2017)). The typical outcome of most of these studies relies on the cell viability following PTT, tumor growth suppression, and/or MoS_2_ clearance after treatment. Indeed, numerous articles report on the biocompatibility and environmental impact of MoS_2_-based nanomaterials (Singh et al. (2020)). However, a definitive and fully comprehensive conclusion has not been reached yet. As a general rule, toxicity depends on several factors, including morphological features (size and shape), surface charge, zeta potential, surface functionalization, and 2D-MoS_2_ amount delivered to cells. Intriguingly, several articles also report on a strong dependence of cytotoxicity by the cell type (Siepi et al. (2017); Shah et al. (2015)), with major effects on tumor cell lines, which hold promise for the concrete use of 2D-MoS_2_ in cancer therapy (Kaur et al. (2018)). The response to 2D-MoS_2_ at the single-cell level is less investigated. Clearly, this kind of information can help in shedding light on the subtle cellular mechanisms ruling the 2D-MoS_2_–cell interaction and, eventually, to 2D-MoS_2_ cytotoxicity (Wu et al. (2016)). This is a fundamental issue for tailoring 2D-MoS_2_ features for biomedical applications.

Optical microscopy techniques are ideally suited to this purpose (Stender et al. (2013)). In particular, confocal fluorescence microscopy has been traditionally considered as the golden standard for optical imaging because of its remarkable sensitivity and sub-micron spatial resolution, giving access to the investigation of subcellular compartments (Sahl et al. (2017)). However, all the fluorescence-based techniques present several drawbacks, which are mainly related to photobleaching and photoblinking of the fluorescent labels (Hu et al. (2016)). Raman spectroscopy (RS) has recently emerged as a reliable and effective technique to overcome these drawbacks (Morrish et al. (2021)). RS is able to provide detailed information about the chemical structure and molecular interactions of the analyte in a label-free, non-destructive approach, thanks to the specific information encoded in Raman spectra (Bergholt et al. (2019)). When applied to the analysis of single cells, RS can provide precious information on the cell biochemistry and its evolution occurring due to cell growth or the interaction with external agents. Recently, these vast potentialities have also been applied to monitor the response of cells when exposed to selected nanoparticles. Particularly, RS has also been applied to investigate the uptake and subcellular localization of MoS_2_ plates (Byrne et al. (2020)). Intriguingly, Raman features corresponding to these particles can also be correlated to the number of layers in the 2D structure and the presence of defects, which has also provided the opportunity to monitor their degradation as a function of time (Moore et al. (2020)). More recently, the localization of 2D-MoS_2_ in specific organelles of U2OS cells has been obtained through volumetric Raman spectroscopy, together with insights into mechanotransduction responses of these cells to substrate-derived nanomaterials (Harries et al. (2021)). Furthermore, Moore et al. (2021) have monitored cellular responses and evolution of organelle compositions in response to exposure to MoS_2_.

In the present article, Raman analysis was used to analyze MCF7 cells interacting with 2D-MoS_2_. In particular, Raman data were used to evaluate both 2D-MoS_2_ localization and temperature increase induced by laser irradiation. For the latter issue, we took advantage of the dependence by the local temperature of the water band in the 2,800- to 3,800-cm^−1^ region, first exploited by Sugimura et al. (2020). With respect to fluorescence-based approaches for temperature measurements at a single-cell level (Okabe et al. (2012)), the Raman-based strategy employed herein is label-free, and it also overcomes the limits of fluorescence related to photobleaching and phot-blinking. This feature is particularly significant for the analysis of the temperature rise in cells upon irradiation with high-intensity lasers, as it happens for testing at the single-cell level, and the use of nanomaterials as PTA (Rivas Aiello et al. (2021)).

Our experimental outcomes demonstrate that laser heating is strongly confined in the region near 2D-MoS_2_ aggregates, which, in turn, tend to occupy the perimembrane region. Temperature rise can reach ∼ 30°C when 2D-MoS_2_ aggregates are irradiated by a laser power ∼ 30 mW in the visible region. Our results pave the way for better comprehension of the effect of PTA on the different subcellular compartments and, more in general, to the cell dynamic leading to cell death following the application of heating protocols.

## 2 Materials and Methods

### 2.1 2D-MoS_2_ Production

#### 2.1.1 Exfoliation of MoS_2_ Powder

The starting commercialized bulk MoS_2_ powder (Sigma Aldrich, 69860, particle size 6 *μ*m, density 5.06 g ml^−1^ at 25°C) was exfoliated in elix water as a pure solvent using a tip sonicator (Bandelin Ultrasound SONOPLUS HD3200, maximum power 200 W, working frequency 20 kHz, KE-76 probe, running at 40*%* amplitude) for 3 h in a cylindrical glass tube (4 cm diameter, 12 cm height and rounded bottom). The temperature of the dispersion during sonication was controlled in an ice water bath. Successive stepwise controlled centrifugation steps were carried out (Eppendorf Centrifuge 5810 R, Rotor F-34-6-38) at different centrifugal forces.

#### 2.1.2 Controlled Centrifugation

Polydispersity in the behavior of exfoliated 2D-MoS_2_ is an important parameter related to the mean lateral nanosheet size ⟨*L*⟩ and number of layers ⟨*N*⟩. The obtained polydisperse 2D-MoS_2_ dispersion was therefore separated into fractions with different *L* and *N* by serial centrifugation steps (Backes et al. (2016)). Details about this procedure are reported in Kaur et al. (2018). In brief, after the first step of centrifugation (total run time = 45 min, g-force = 100 g), the sediment (mainly containing unexfoliated 2D-MoS_2_) was discarded, whereas the supernatant was re-dispersed into a fresh solvent and shifted to the next centrifugation step for treatment at higher centrifuge speed. By the end of this serial procedure, the supernatant was found to contain thinner and smaller nanoflakes. The analysis shown in this article is based on samples obtained at a g-force = 1,000 g in the final step. Before use, 2D-MoS_*s*_ dispersion was characterized by *1*) UV–visible spectroscopy, *2*) light dynamic scattering (LDS) and *ζ* potential, and *3*) Raman.

### 2.2 Raman Setup

Raman measurements were carried out using the commercial confocal micro-Raman system WiTec Alpha 300. It consists of an integrated system, combining an inverted confocal Raman microscope with an AFM system (placed on the top of the inverted microscope). The Raman microscope was endowed with a probe at 532 nm, provided by a frequency-doubled Nd-YAG laser. The Raman beam was focused on the sample through a 100X objective (Olympus, PlanApo, oil immersion, NA = 1.4). Raman photons were collected in backscattering geometry by using the same focusing objective lens. After passing through an edge filter, such radiation was directed into the spectrometer through a 50-*μ*m core fiber, which also acts as a pinhole for confocal detection. The Raman radiation was detected using a thermoelectrically cooled charge-coupled device at the spectrometer exit. The in-plane and axial resolution (PSF-HWHM) were ∼ 0.3 and 1 *μ*m, respectively, measured following Cai et al. (1998). Typical powers impinging on the sample were of the order of a few milliwatts which, in the focal region, correspond to laser intensities of the order of 10^6^ W/cm^2^. Raman measurements were performed on 2D-MoS_2_ adhered on 170-*μ*m-thick glass coverslips. In the case of cells, glass-bottom imaging chambers (Ibidi, glass-bottom *μ*-dishes) were used.

### 2.3 Cell Line Culture and Treatment

MCF7 (human breast adenocarcinoma cell line) cells were grown in DMEM supplemented with 10*%* heat-inactivated FBS (Sigma Aldrich), 1*%* glutamine (Euro Clone), 1*%* penicillin/streptomycin (EuroClone), and 0.1*%* gentamycin (EuroClone), at 37°C in air containing 5*%* CO_2_. MCF7 cells were cultured for 24 h prior to treatment. After that, a volume of 100 *μ*L of the sonicated MoS_2_ solution at a concentration of 0.12 mg/ml was added to the cell plate (4 × 10^3^ cells/well), and the incubation was carried out for 24 h. The cells were fixed with 2.5*%* glutaraldehyde in 0.2 M PBS at pH 7.2–7.4 for 2–4 h at 4°C. The cells were then washed three times for 10 min with 0.2 M PBS, which was the final mounting medium. An observation at the optical microscope showed that most of 2D-MoS_2_ had been removed, except for a few of them, which, as discussed later in the article, remained adhered to the external surface of the cells.

## 3 Results and Discussion

### 3.1 2D-MoS_2_ Characterization

#### 3.1.1 UV–Visible Spectrum

**FIGURE 1:**
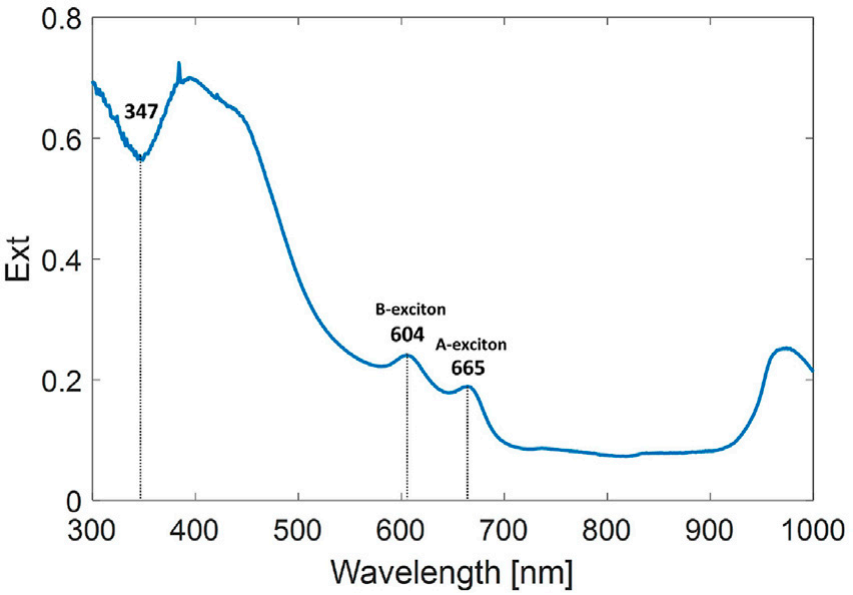
UV–visible extinction spectrum of 2D-MoS_2_ solution centrifuged at 1,000 g.

Optical extinction spectra were acquired on a Perkin Elmer Lambda 35 UV–Vis spectrophotometer using a 1-cm-thick quartz cuvette. The extinction spectrum of the 2D-MoS_2_ dispersion used in this study is shown in Figure 1. From the ratio of extinction at B-exciton to that at 347 nm, *Ext*_*B*_/*Ext*_347_, and the peak position of A-exciton, *λ*_*A*_, we can estimate ⟨*N*⟩∼4 layers and ⟨*L*⟩ ∼123 nm by using the metrics, as explained in Backes et al. (2014).

#### 3.1.2 DLS and *ζ*-Potential Measurements

**FIGURE 2:**
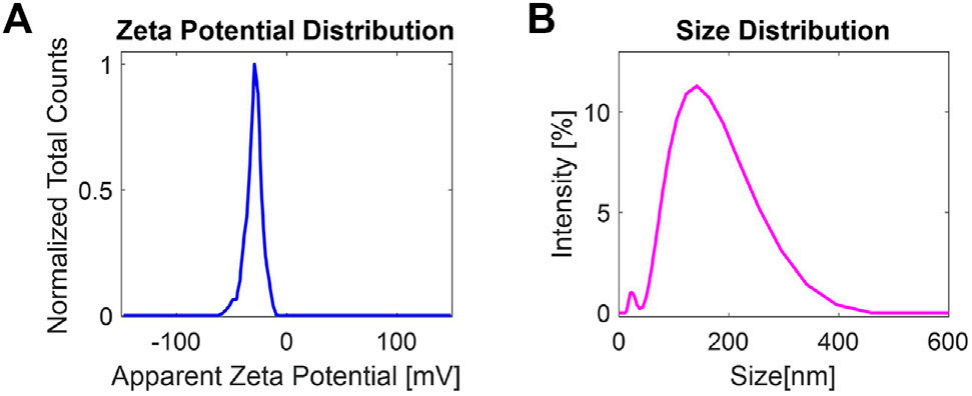
**(A)** *ζ*-Potential distribution and **(B)** size distribution of 2D-MoS_2_ produced at a g-force of 1,000 g.

Liquid-phase exfoliation produces surface charges over the surface of exfoliated 2D-MoS_2_, which plays an important role in understanding the stability of liquid-exfoliated dispersions. Size and *ζ*-potential distribution measurements were carried out by using a Zetasizer equipped with a He–Ne laser (Malvern Zetasizer Nano system). All the measurements were carried out at 25°C within a few hours from the exfoliation. Figure 2A shows the *ζ*-potential distribution of exfoliated 2D-MoS_2_, exhibiting an average zeta potential ∼−29.8 mV. At this *ζ*-potential value, the dispersion can be considered stable. Figure 2B, instead, depicts the size distribution of the nanoflakes dispersed in pure water. The obtained mean size, taken as the average of three runs measured at 25°C in disposable folded capillary cells (DTS1070), is ∼ 140 nm, which is in good agreement with the average size ⟨*L*⟩ estimated from the previously UV–Vis measurements.

##### 3.1.3 Micro-Raman spectroscopy of 2D-MoS_2_

Raman spectroscopy is a widely employed tool to estimate the thickness of TMD nanoflakes (Kaur et al. (2017a); Lee et al. (2010); Zhang et al. (2015); Kaur et al. (2017b)). The Raman spectrum of MoS_2_ shows characteristic bands, $E_{2g}^{1}$ and *A*_1*g*_, corresponding to in-plane and out-of-plane vibrational modes, for bulk fall at about 380 cm^−1^ and 403 ^−1^, respectively. MoS_2_ nano-structuring modifies the Raman features of the bulk with an increase in the $E_{2g}^{1}$ frequency and a corresponding decrease in the *A*_1*g*_.

$$\Delta \nu_{MoS_{2}}=\nu_{A_{1g}}-\nu_{E_{2g}^{1}}.$$ (1)

**FIGURE 3:**
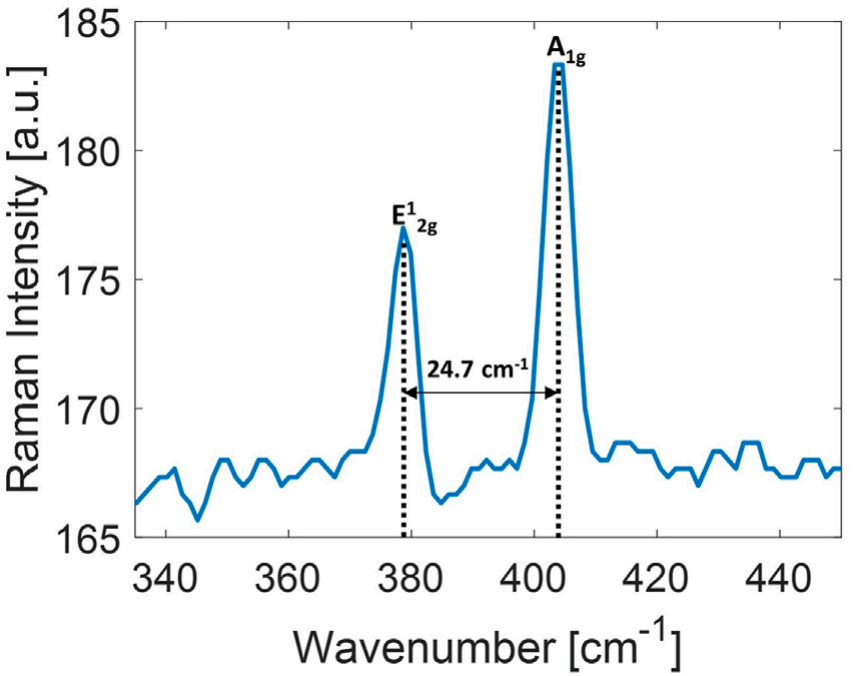
Raman spectrum of 2D-MoS_2_ dispersion employed in this study. The distance between the two main peaks of MoS_2_ modes is also indicated.

The resulting frequency shift identifies the number of layers in the nanoflakes. A typical spectrum of 2D MoS_2_ nanoflakes centrifuged at 1,000 g is shown in Figure 3. The $\Delta \nu_{MoS_{2}}$ range observed via micro-Raman spectroscopy corresponds to a nano-structuring spanning from 3 to 4 layers. This outcome is consistent with the range of nano-structuring indicated by UV–Vis extinction spectroscopy.

### 3.2 Heating at Microscale: Preliminary Considerations and Experimental Design

Laser heating of samples induced by the presence of PTA depends on three important factors: *1*) heating laser intensity *I*, *2*) PTA absorption at the laser wavelength, *3*) PTA amount in the sample. Moreover, in order to analyze the thermal response of systems to laser irradiation, it is important to properly take into account the length scales involved in the process (Qin and Bischof (2012)). For instance, by considering a *d*-sized system loaded with PTA at a number density *N* (NPs/m^3^), the laser intensity (W/m^2^) needed to get a given temperature increase Δ*T* (K) is ruled by the equation (Qin and Bischof (2012)):

$$\Delta T=\frac{N\cdot d^{2}\cdot C_{abs}\cdot I}{2k},$$ (2)

where *C*_*abs*_ is the PTA absorption cross section and *k* (W/m K) the thermal conductivity of the medium in which the system lies. According to the *d* ^2^ dependence shown in Eq. 2, the laser intensity required to produce a given temperature increase in a single cell (d ∼ 10 *μ*m) placed in an infinite aqueous medium is ∼ 10^6^ times higher than that required to get the same temperature rise in a 10-mm tumor. Therefore, while PTT in macroscopic systems requires laser intensities of the order of a few W/cm^2^ or less (Wang et al. (2021)), *I* values in the order of 10^6^ W/cm^2^ are required to simulate thermal treatment at the single-cell level. A similar scaling holds in the time domain (Qin and Bischof (2012)). As a matter of fact, the characteristic time *τ*_*T*_, that is, the time needed to reach thermal equilibrium with the surrounding medium by a *d*-sized absorbing system, scales according to the relation:

$$\tau_{T}=\frac{d^{2}}{\alpha },$$ (3)

where *α* is the medium thermal diffusivity. Considering, for instance, the case of a cell with a 10 *μ*m diameter and using the thermal diffusivity of water (0.143 mm^2^/s at room temperature), the characteristic time is *τ*_*T*_∼ 0.7 ms. On the other hand, *τ*_*T*_ is in an order of a few minutes for *R* = 10 mm, which is consistent with the typical duration of a PTT on tissues (Chou et al. (2013); Carrow et al. (2020); Liu et al. (2021); Song et al. (2017)).

Considering these scaling laws, some important issues can be highlighted. First, it is worth noticing that laser intensities required to heat single PTA-loaded cells (∼ 10^6^ W/cm^2^) are reached by focusing laser beams with a power of a few tens of mW with high NA microscope objectives, similar to typical micro-Raman experiments, where the laser spot is ∼λ . Second, in single-cell experiments, with *τ*_*T*_ in the ms range, it results much shorter than the integration time *τ* typically used for the acquisition of Raman spectra (0.1–10 s range). Accordingly, in our analysis, it can be safely assumed that the temperature captured by Raman analysis corresponds to a steady-state condition. Taken together, these issues allow the use of the same Raman laser for both sample irradiation (*irradiation* laser) and temperature estimation (*probe* laser).

### 3.3 Temperature Calibration by Water Band

**FIGURE 4:**
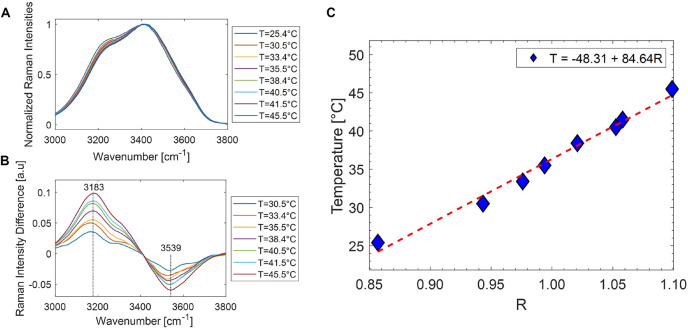
**(A)** Raman water bands in the 3,000- to 3,800-cm^−1^ spectral range, normalized to the prominent feature at 3,400 cm^−1^, obtained at different temperatures. When temperature increases, the low wavenumber side decreases accordingly. **(B)** Difference in the spectra reported in part A with respect to the corresponding one at 25.4°C. **(C)** Trend of the ratio *R* *vs.* T, together with the line corresponding to a linear fitting of the data. The calibration line equation is also indicated.

In order to assess the sample temperature by Raman analysis of the OH band, a preliminary calibration was performed by acquiring the Raman spectra of distilled water taken at controlled temperature. To this purpose, a glass cell filled with water was put in contact with a thermo-electric Peltier cell, driven by a temperature controller. The water temperature, monitored by an NTC probe, was stabilized within 0.1°C. Therefore, we acquired the water Raman band in the 3,000- to 3,800-cm^−1^ region as a function of the temperature in the 23°C–45°C range. Figure 4A reports the acquired spectra, normalized at the prominent band at ∼ 3,400 cm^−1^, while Figure 4B shows the difference of spectra acquired at each temperature T with respect to that acquired at room temperature (T = 25.4°C). Clearly, as temperature increases, the higher wavenumber side (asymmetric stretching) of the spectra increases and the lower wavenumber wing (symmetric stretching) decreases. According to Khoshtariya et al. (2003), this is consistent with a weakening of the hydrogen bonds among the interacting water molecules in the solution. In particular, the highest band variations occur around 3,183 and 3,539 cm^−1^, so that the ratio

$$R=\frac{I_{3539}}{I_{3183}}$$ (4)

is quite sensitive to temperature. Figure 4C reports the *R* values obtained at the different analyzed temperatures. The error bar on each experimental determination is ∼ 0.8°C. The linear trend observed suggests the use of *R* as a reliable parameter to monitor the local temperature in an aqueous environment. Therefore, a linear fit of the data was performed, and the obtained best-fit parameters (shown in the label of Figure 4C) were used for the successive sample temperature measurements. Notably, the obtained line parameters are consistent with those reported in Sugimura et al. (2020) for cell medium in the 25°C–45°C range.

### 3.4 Temperature Mapping of 2D-MoS_2_

**FIGURE 5:**
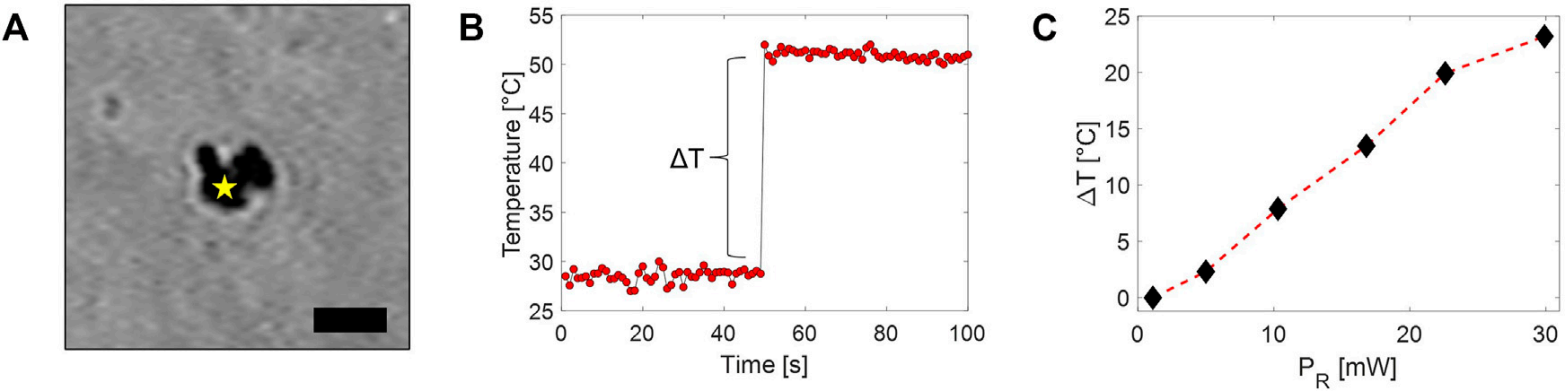
**(A)** Bright-field image of a 2D-MoS_2_ agglomerate adhered to a glass coverslip (scale bar = 2 *μ*m). The star highlights the position of the Raman probe during the acquisition of the time series of Raman spectra, leading to temperature data shown in part B. **(B)** Temperature trend of the 2D-MoS_2_ agglomerate obtained under irradiation of the Raman beam. The sharp temperature increase is obtained in correspondence of the *P*_*R*_ stepwise increase. **(C)** Measured 2D-MoS_2_ agglomerate temperatures rise observed under irradiation at different *P*_*R*_.

In order to explore the effectiveness of the Raman-based approach for temperature mapping, we first analyzed the thermal response of 2D-MoS_2_ in an aqueous environment. For this purpose, we deposited a drop of about 20 *μ*L of 2D-MoS_2_ aqueous dispersion on a coverslip. During the evaporation, 2D-MoS_2_ nanoflakes tend to aggregate because of their hydrophobicity. Indeed, once the drop evaporated, small agglomerates of nanoparticles of a few microns size were observed on the slide (see Figure 5A). Then, a sandwiched cell was prepared by gluing a second coverslip using two parafilm strips as spacers. The created small chamber was filled with distilled water and sealed off. Therefore, we acquired a time series of Raman spectra in a point spatially close to the 2D-MoS_2_ agglomerate. In particular, each spectrum was acquired by using an integration time of 1 s, while the Raman probe power *P*_*R*_ was kept to 2 mW for the first 50 s, whereupon it was instantaneously increased to 30 mW for the following 50 s. Finally, the local 2D-MoS_2_ temperature was estimated according to the calibration line previously found. The obtained temperature values are reported in Figure 5B. Interestingly, these data exhibit a stepwise behavior, with a sudden temperature increase of ∼ 25° occurring as a result of the *P*_*R*_ stepwise increase. This clearly means that a temperature equilibrium between the irradiated agglomerate and the surrounding environment is rapidly reached. Intriguingly, the relatively short temperature rising time (which is not appreciable with our sampling rate) is consistent with a *τ*_*T*_ in the ms range that can be reasonably expected for our micro-size system.

Similar measurements have been repeated by acquiring several time series of Raman spectra at different *P*_*R*_. The local temperature rise estimated from these spectra is reported in Figure 5C. As expected, a linear trend was observed, with a possible beginning of saturation for high *P*_*R*_ values.

**FIGURE 6:**
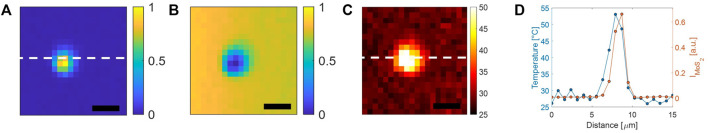
**(A)** Raman maps corresponding to $I_{MoS_{2}}$ obtained by raster scanning in a 15-*μ*m × 15-*μ*m region (scale bar = 4 *μ*m). This map highlights the localization of the 2D-MoS_2_ agglomerate. **(B)** Intensity *I*_*w*_ of the integrated water Raman band in the 3,000- to 3,800-cm^−1^ spectral region obtained in the same region analyzed in part A. **(C)** Temperature map obtained starting from the estimation of the *R* ratio in the scanned region. **(D)** Comparison of $I_{MoS_{2}}$ and *T* values obtained along the white cross-sectional lines highlighted in parts A and C.

Finally, measurements were performed in order to estimate the localization of the temperature increase induced by laser heating. For this purpose, we acquired a Raman map by raster scanning in a 15-*μ*m × 15-*μ*m region in a plane at a height ∼ 1 *μ*m from the glass coverslip. In particular, spectra were acquired with *P*_*R*_ = 30 mW and *τ* = 1 s. Figure 6 reports both $I_{MoS_{2}}$ , the intensity of the sharp MoS_2_ peak at ∼ 400 cm^−1^ (part A), and *I*_*w*_, the intensity of the integrated water Raman band in the 3,200- to 3,800-cm^−1^ spectral region (Figure 6B). Clearly, the first map gives evidence of the presence of a 2D-MoS_2_ agglomerate, which, by steric effect, produces a water band intensity lowering in the second map. Therefore, starting again from the calibration curve of Figure 4C, it was possible to reconstruct the temperature profile in the scanned region. Figure 6C reports the obtained thermal map. Interestingly, the thermal map reveals a significant temperature rising only in the region localized very close to the 2D-MoS_2_ agglomerate. The co-localization of 2D-MoS_2_ and temperature rise are better appreciated in Figure 6D, where we compare $I_{MoS_{2}}$ and *T* values, along the cross-sectional lines highlighted in Figure 6A. It is also worth noticing that the estimated surrounding temperature (27°C) is consistent with room temperature. This issue is clearly explained by the negligible absorption of water in the *green* region.

### 3.5 Analysis of the Photothermal Effect Induced in MCF-7 Cells by 2D-MoS_2_

Once we assessed the effectiveness of the Raman-based approach for revealing the heat release by 2D-MoS_2_ upon irradiation by lasers in an aqueous environment, we analyzed 2D-MoS_2_ as PTA acting on MCF7 cells.

Temperature profiling inside cells by using the previously illustrated Raman approach is trickier, due to the presence of different concomitant effects able to alter the *R* ratio independently from temperature. It is in fact well known that *R* can be modified by the presence of salts, and, more in general, by the complex water molecules environment, as found in Puppulin et al. (2017). Moreover, some interferences could be, in principle, expected from bands assignable to other O–H groups in organic macromolecules present in cells. In this regard, however, it is worth noticing that O–H bands in cells are expected to be dominated by water contribution, water being the main constituent of cells. This is obviously in line with the relatively weak dependence of the *R vs. T* calibration line parameters obtained previously in different cellular compartments (Sugimura et al. (2020)).

**FIGURE 7:**
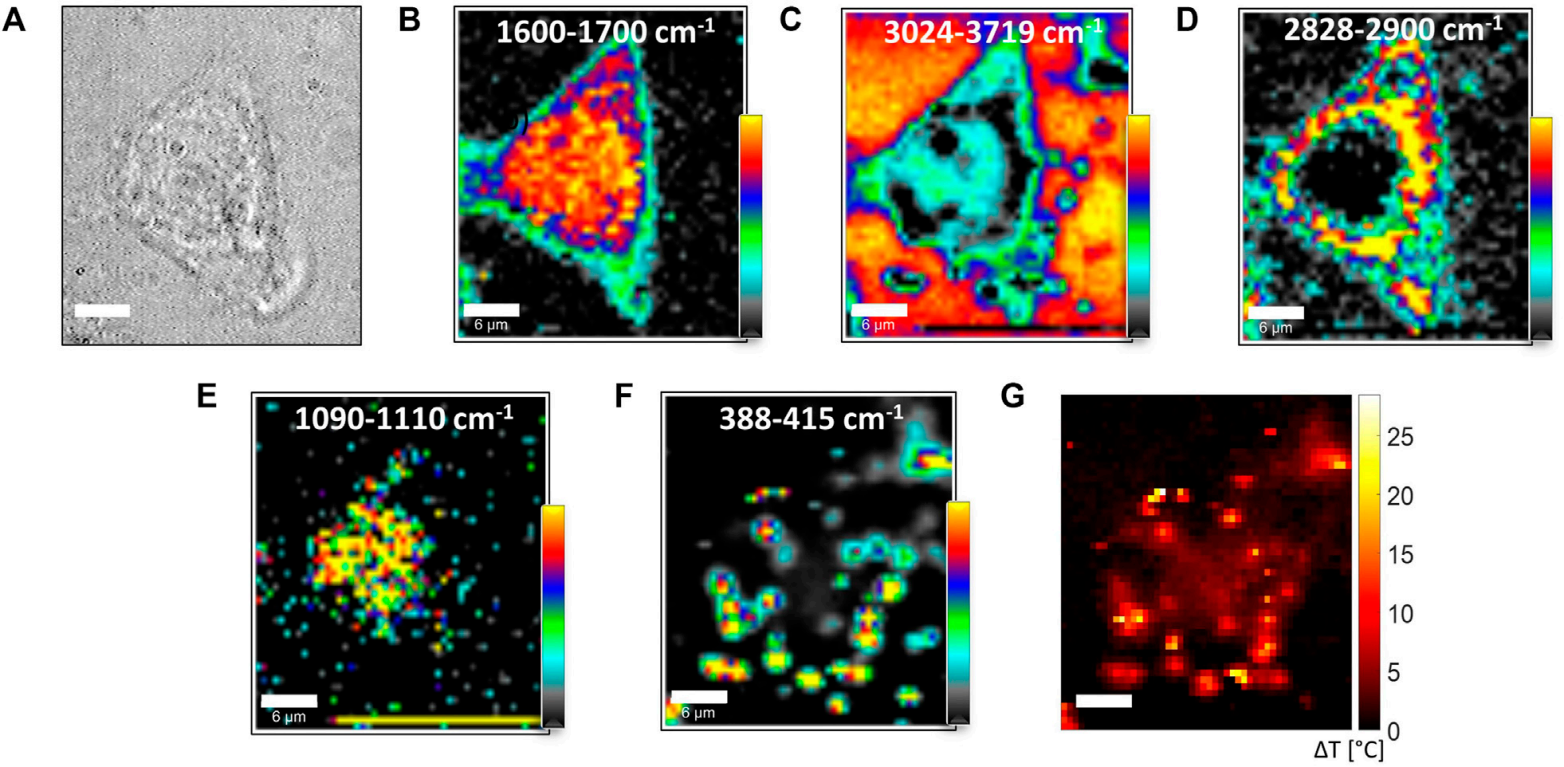
**(A)** Bright-field image of an MCF7 cell exposed to 2D-MoS_2_. **(B–F)** Integrated intensity of selected bands obtained in a raster scanning of a 30-*μ*m × 30-*μ*m region with a 600-nm step. *P*_*R*_ and *τ* were 30 mW and 2 s, respectively, so the whole image was acquired in ∼ 83 min. Confocal Raman section was acquired in a plane, cutting through the nucleus, at ∼ 5 *μ*m from the glass interface. The labels in each panel correspond to the spectral regions selected for band intensity integrations. Color scales are relative scales, with the darker and brighter colors corresponding to the lowest and highest integrated intensity values, respectively. Such maps provide information on the spatial distribution of proteins (panel B), water (panel C), lipids (panel D) DNA (panel E), and 2D-MoS_2_ (panel F). **(G)** Temperature increase of the analyzed cell obtained starting from the estimation of the *R* ratio in the scanned region.

In our experiment, in order to single out the role of temperature in ruling the shape of O–H features in the 3,000- to 3,800-cm^−1^ region, we took advantage of the fact that while *R* changes related to the variation of the local O–H bond environment can be safely assumed to be independent of *P*_*R*_, a linear dependence by *P*_*R*_ can be instead observed for *R* changes related to heating. Starting from this observation, it can be easily argued that *R*-maps measured at different Raman *P*_*R*_ exhibit appreciable variations only in points where heating is produced by the probe beam itself. As a result, Δ*R* maps, that is, the difference of maps obtained starting from different *P*_*R*_, can be correlated to the local temperature rise Δ*T* induced by laser heating. This differential approach was used to get the Δ*T* map of a whole cell targeted with 2D-MoS_2_ upon irradiation by a laser beam. The outcomes of this analysis are reported in Figure 7. In particular, Figure 7A shows the bright-field cells’ image, while Figures 7B–F report the integrated intensities of Raman bands in the spectral regions indicated in each figure label. Such maps highlight the spatial distribution of proteins (panel B), water (panel C), lipids (panel D), DNA (panel E), and 2D-MoS_2_ (panel F). An analysis, in terms of the number of layers of 2D-MoS_2_ aggregates, is reported in Supplementary Figure S1. From the map of panel D, it is possible to distinguish the nuclear membrane closing the nucleus, which is, in turn, highlighted by DNA bands in panel E. The cell edge can be, instead, well identified from the protein map and water map, resulting in this latter as a water loss. In order to better appreciate the co-localization of selected macromolecules, some merge images have been created and reported in the Supporting Material (Supplementary Figure S2). Intriguingly, 2D-MoS_2_ seem to remain confined in the proximity of membranes, suggesting that 2D-MoS_2_ aggregates are not able to penetrate the cell. In this regard, it is worth noticing that previous studies have clearly demonstrated that dispersed 2D-MoS_2_ are internalized by cells through three different endocytosis pathways, and autophagy mediates their accumulation in lysosomes (Zhu et al. (2018)). Other studies (Moore et al. (2020)) revealed the presence of both phosphatidyl lipid vesicles and lysozymes in the microenvironment of internalized 2D-MoS_2_. In this case, lysozymes were associated with MoS_2_ degradation. Nevertheless, as a general rule, the internalization capability of cells and NP fate are strongly affected by particle size, and it is strongly reduced for larger particles (and particles aggregates, as in our experiment), which tend to remain outside the cells. This could clearly explain our outcome. Clearly, a more definitive conclusion regarding this issue could be reached by 3D confocal scans on a statistically significant number of cells. However, this study is out of the scope of the present investigation.

Heating induced by the Raman probe was evaluated by the data obtained with P_*R*_ = 2 mW and P_*R*_ = 30 mW, according to the procedure described before. The resulting Δ*T* map is reported in 7G. Intriguingly, high Δ*T* values are obtained in correspondence of 2D-MoS_2_ aggregate positions, giving evidence of the strong confinement of the heating induced by laser irradiation. In particular, Δ*T* reaches ∼ 25°C for 2D-MoS_2_ aggregates. It is worth underlining that in many studies, photothermal effects induced by 2D-MoS_2_ are performed using near-IR radiation (808 nm). Such radiation falls within the NIR–water window, where water absorption is negligible. Therefore, such radiation easily penetrates living organisms, which renders it ideally suited for *in vivo* studies and photothermal applications. On the other hand, visible light is strongly absorbed by hemoglobin, so its use is avoided in PTT treatments. However, such issues do not affect our experimental outcomes, which are mainly aimed to reveal the thermal gradient inside and outside single cells targeted with 2D-MoS_2_. It should be finally noticed that in absolute terms, the temperature increase shown in Figure 7G could be in principle achieved by irradiating the sample with an IR laser (usually employed in PTTs) by simply scaling the impinging power *P*_*IR*_ on the sample according to the relation:

$$P_{IR}=\frac{C_{abs_{532}}}{C_{abs_{IR}}}\cdot P_{R}.$$ (5)

For instance, according to the extinction curve reported in Figure 1, the same temperature increase could be achieved by using a laser at 808 nm at a power *P*_*IR*_ ∼100 mW.

Finally, some general considerations can be made on the possibility to extend the Raman-based temperature measurements to *in vivo* experiments. For such studies, in order to provide easy access to the selected anatomical locations, fiber-coupled Raman probes are usually required (“endoscopic” Raman probes). Typically, NIR Raman probes at powers in the 10–150 mW range are used. They are focused down to focal spot diameters ∼ 0.2 mm, hence producing an intensity in the 10–10^2^ W/cm^2^ range (Cordero et al. (2018)). In such conditions, no cellular damage is usually observed, due to the very low absorption of cells in this region. Therefore, endoscopic Raman systems could be safely employed for local temperature measurements. For instance, it could be used to monitor the temperature rise in healthy tissue in the proximity of PTA-targeted tissues under laser irradiation. Such measurement could help to optimize the PTT treatment parameters such as laser fluence, PTA dose, and tissue exposure time.

## 4 Conclusion

In this work, we have shown that micro-Raman spectroscopy is a powerful tool to monitor, at the single-cell level, the photothermal effect induced by optical absorption of 2D-MoS_2_ aggregates. The spectroscopic observable for temperature measurements was the water band around 3,400 cm^−1^, which corresponds to the overlapping of O–H symmetric and asymmetric stretching vibrational modes. This band is altered when the hydrogen bonds involved in the well-known tetrahedral structure of water are perturbed either by an increase in temperature or by the O–H chemical environment (salts, proteins, etc.). In our experiment, the same laser used to induce heating was used as a probe to acquire Raman spectra. Our results demonstrate that laser irradiation of cells targeted with 2D-MoS_2_ leads to a significant temperature increase in a region localized around the 2D-MoS_2_ aggregates. Moreover, Raman maps also denote that these agglomerates seem to remain confined in the proximity of membranes, suggesting that they are not able to penetrate cells. Our results also lay the groundwork for further studies for a deeper understanding of the effects of 2D-MoS_2_ on cells. In fact, having established that 2D-MoS_2_ aggregates tend to be accumulated on the external part of the cell membrane, heating could have a direct effect on the complex structure of the membrane itself, which, in addition to the phospholipid bilayer, consists of a large variety of proteins that regulate exchanges between the internal and external parts of the cell. From this point of view, we envisage the possibility to combine temperature information with information obtained from spectra in the so-called fingerprint region in order to investigate the evolution of selected macromolecules (lipid, proteins, etc.) under thermal treatment.

## Data Availability

The raw data supporting the conclusion of this article will be made available by the authors, without undue reservation.
