# Supplementary figures and images for: Single-Cell Photothermal Analysis Induced by MoS2 Nanoparticles by Raman Spectroscopy
Source: Front Bioeng Biotechnol. 2022 Mar 10;10:844011. doi: 10.3389/fbioe.2022.844011 (PMC8960122; doi:10.3389/fbioe.2022.844011)

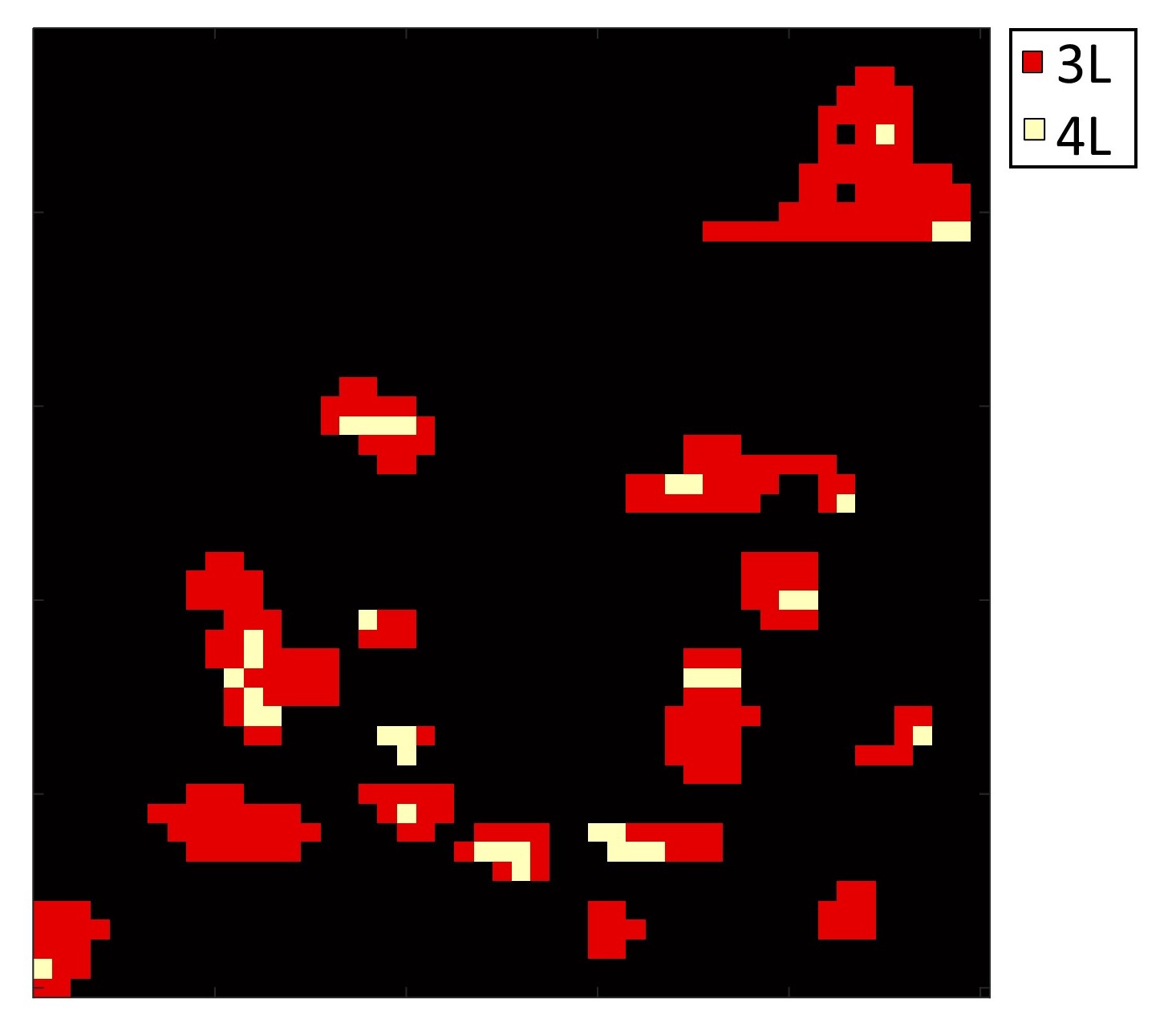

Supplement: Supplementary file 1 [file Image1.JPEG]

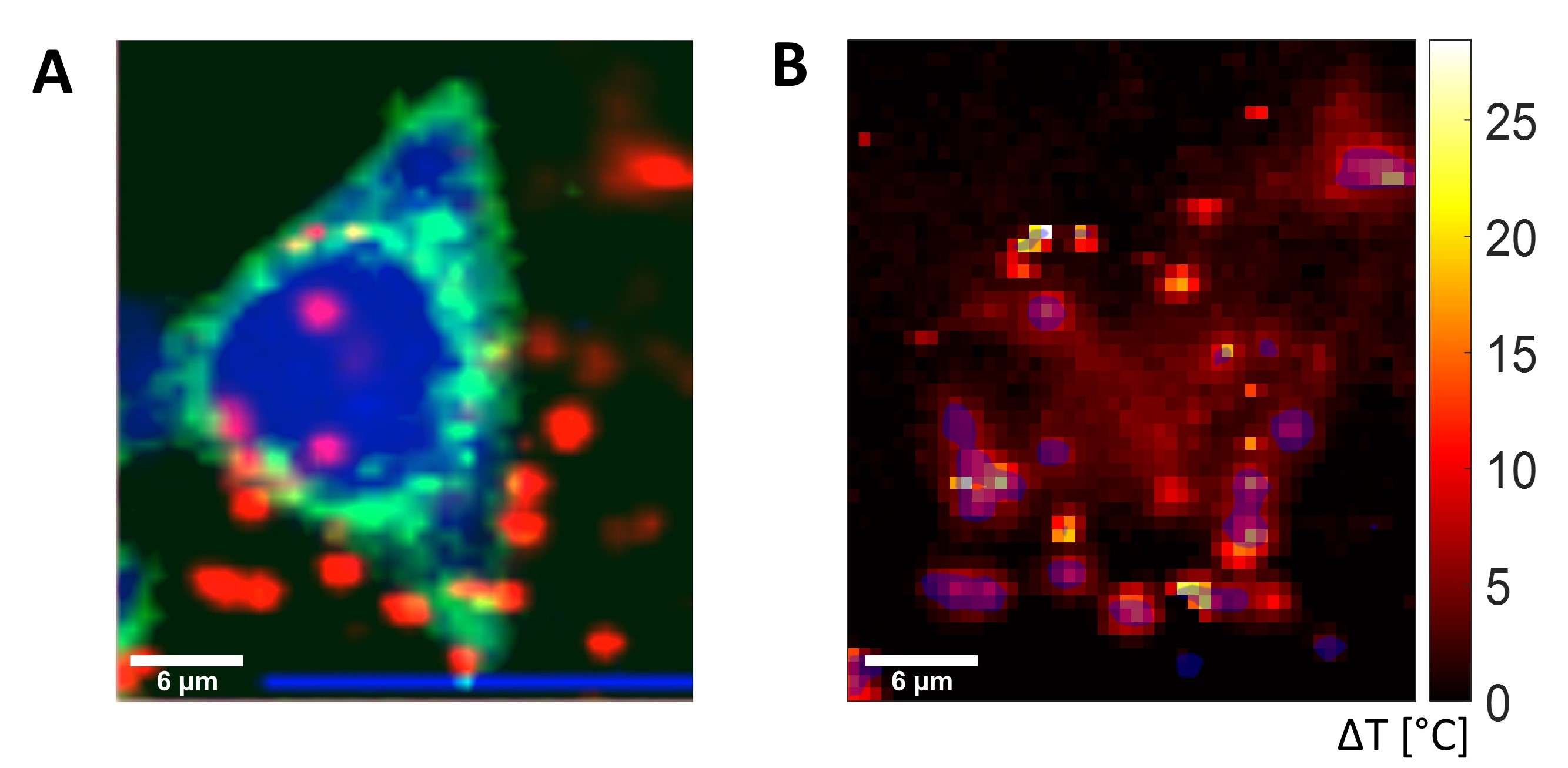

Supplement: Supplementary file 2 [file Image2.JPEG]
